# Supplementary material for: Prevalence and appropriateness of in-person versus not-in-person ambulatory antibiotic prescribing in an integrated academic health system: A cohort study
Source: PLoS One. 2023 Jul 27;18(7):e0289303. doi: 10.1371/journal.pone.0289303 (PMC10374053; doi:10.1371/journal.pone.0289303)
Supplement: Attachment — Submitted filename: PONE-D-23-01039R1, Response to Reviewers.docx [file pone.0289303.s003.docx]

**Journal Requirements**	NB: Page and Line Numbers are from revised, tracked changes revision

| **Page** | **Line** | **Comment** |
| --- | --- | --- |
| General Comment | | *1. Please ensure that your manuscript meets PLOS ONE's style requirements, including those for file naming. The PLOS ONE style templates can be found at*  [*https://journals.plos.org/plosone/s/file?id=wjVg/PLOSOne_formatting_sample_main_body.pdf*](https://urldefense.com/v3/__https:/journals.plos.org/plosone/s/file?id=wjVg*PLOSOne_formatting_sample_main_body.pdf__;Lw!!Dq0X2DkFhyF93HkjWTBQKhk!XlQEZ-eBiwT6sSz-kqrogE90c_NFbv7LH-myuC8pwbDuKKIiqyEQlo0bf1t3fhsDOaOc2sA4CdbAEUSSQdFhOw$) *and*  [*https://journals.plos.org/plosone/s/file?id=ba62/PLOSOne_formatting_sample_title_authors_affiliations.pdf*](https://urldefense.com/v3/__https:/journals.plos.org/plosone/s/file?id=ba62*PLOSOne_formatting_sample_title_authors_affiliations.pdf__;Lw!!Dq0X2DkFhyF93HkjWTBQKhk!XlQEZ-eBiwT6sSz-kqrogE90c_NFbv7LH-myuC8pwbDuKKIiqyEQlo0bf1t3fhsDOaOc2sA4CdbAEURcOBeGTw$)  We have reviewed the PLOS ONE style requirements in the cited documents and revised the manuscript, including changing the header fonts, moving the Tables into the main text, reorganizing the tables per PLOS ONE style, and changing the file name of the Supporting Information. |
| 20 | 371 | *2. Thank you for stating the following in the Acknowledgments Section of your manuscript: "This research was supported by grant number R01HS024930 from the Agency for Healthcare Research and Quality (AHRQ). The contents of this product are solely the responsibility of the authors and do not necessarily represent the official views of or imply endorsement by AHRQ or the U.S. Department of Health and Human Services. The funders had no role in study design, data collection or analysis, decision to publish, or preparation of the manuscript."*  *We note that you have provided funding information that is not currently declared in your Funding Statement. However, funding information should not appear in the Acknowledgments section or other areas of your manuscript. We will only publish funding information present in the Funding Statement section of the online submission form.*   *Please remove any funding-related text from the manuscript and let us know how you would like to update your Funding Statement. Currently, your Funding Statement reads as follows:*   *"This research was supported by grant number R01HS024930 from the Agency for Healthcare Research and Quality (AHRQ). The contents of this product are solely the responsibility of the authors and do not necessarily represent the official views of or imply endorsement by AHRQ or the U.S. Department of Health and Human Services. The funders had no role in study design, data collection or analysis, decision to publish, or preparation of the manuscript."*  *Please include your amended statements within your cover letter; we will change the online submission form on your behalf.*  We have removed funding from the Acknowledgement. The funding statement should read:  “This research was supported by grant number R01HS024930 from the Agency for Healthcare Research and Quality (AHRQ). Dr. Linder is also supported by grants from the National Institute on Aging (P30AG059988, R01AG069762, R01AG074245, P30AG024968, R01AG070054, R33AG057395) and the Agency for Healthcare Research and Quality (R01HS026506, R01HS028127). The contents of this product are solely the responsibility of the authors and do not necessarily represent the official views of or imply endorsement by AHRQ or the U.S. Department of Health and Human Services. The funders had no role in study design, data collection or analysis, decision to publish, or preparation of the manuscript.”  Also, on reviewing the PLOS ONE submission guidelines, we see there is no need for a Contributor and Guarantor Information, which we have removed. |
| General Comment | | *3. In your Data Availability statement, you have not specified where the minimal data set underlying the results described in your manuscript can be found. PLOS defines a study's minimal data set as the underlying data used to reach the conclusions drawn in the manuscript and any additional data required to replicate the reported study findings in their entirety. All PLOS journals require that the minimal data set be made fully available. For more information about our data policy, please see* [*http://journals.plos.org/plosone/s/data-availability*](https://urldefense.com/v3/__http:/journals.plos.org/plosone/s/data-availability__;!!Dq0X2DkFhyF93HkjWTBQKhk!XlQEZ-eBiwT6sSz-kqrogE90c_NFbv7LH-myuC8pwbDuKKIiqyEQlo0bf1t3fhsDOaOc2sA4CdbAEUSL2w0oGA$)*.*  *"Upon re-submitting your revised manuscript, please upload your study’s minimal underlying data set as either Supporting Information files or to a stable, public repository and include the relevant URLs, DOIs, or accession numbers within your revised cover letter. For a list of acceptable repositories, please see* [*http://journals.plos.org/plosone/s/data-availability#loc-recommended-repositories*](https://urldefense.com/v3/__http:/journals.plos.org/plosone/s/data-availability*loc-recommended-repositories__;Iw!!Dq0X2DkFhyF93HkjWTBQKhk!XlQEZ-eBiwT6sSz-kqrogE90c_NFbv7LH-myuC8pwbDuKKIiqyEQlo0bf1t3fhsDOaOc2sA4CdbAEUTznPVmhA$)*. Any potentially identifying patient information must be fully anonymized.*  *Important: If there are ethical or legal restrictions to sharing your data publicly, please explain these restrictions in detail. Please see our guidelines for more information on what we consider unacceptable restrictions to publicly sharing data:* [*http://journals.plos.org/plosone/s/data-availability#loc-unacceptable-data-access-restrictions*](https://urldefense.com/v3/__http:/journals.plos.org/plosone/s/data-availability*loc-unacceptable-data-access-restrictions__;Iw!!Dq0X2DkFhyF93HkjWTBQKhk!XlQEZ-eBiwT6sSz-kqrogE90c_NFbv7LH-myuC8pwbDuKKIiqyEQlo0bf1t3fhsDOaOc2sA4CdbAEUSbGzM74w$)*. Note that it is not acceptable for the authors to be the sole named individuals responsible for ensuring data access.*  *We will update your Data Availability statement to reflect the information you provide in your cover letter.* With apologies, we updated the Data Availability statement to the following:  Restrictions apply to the availability of these data because they are potentially identifiable, sensitive, protected health information of Northwestern Medicine patients. Making the data externally available requires the approval of the Northwestern Medicine Data Steward and Privacy Official, Rayan Venkatesh, Director of Integrity, Corporate Compliance and Integrity (email, rvenkate@nm.org; phone, 312-695-5913).  By way of explanation, Northwestern Medicine is a separate corporate entity from Northwestern University. |
| 25 | 506 | *4. Please include captions for your Supporting Information files at the end of your manuscript, and update any in-text citations to match accordingly. Please see our Supporting Information guidelines for more information:* [*http://journals.plos.org/plosone/s/supporting-information.*](https://urldefense.com/v3/__http:/journals.plos.org/plosone/s/supporting-information.__;!!Dq0X2DkFhyF93HkjWTBQKhk!XlQEZ-eBiwT6sSz-kqrogE90c_NFbv7LH-myuC8pwbDuKKIiqyEQlo0bf1t3fhsDOaOc2sA4CdbAEUS7XznZNg$)  We have included captions for our Supporting Information files and updated the in-text citations so that the naming is consistent in the text, captions, and the file itself. |

**Reviewer 1 Comments**	NB: Page and Line Numbers are from revised, tracked changes revision

| **Page** | **Line** | **Comment** |
| --- | --- | --- |
| General Comment | | *Thank you for the chance to review this manuscript, which examines antibiotics prescribed in outpatient settings in an integrated health delivery system in the US, detailing those prescribed with in-person visits versus outside of in-person visit. They found that 19% of antibiotics were prescribed outside of in-person visits (primarily telephone visits). Compared to antibiotics prescribed with an in-person visit, those prescribed outside of an in-person visit were more likely to be chronic or to lack an associated diagnosis.*  We appreciate this summary from Reviewer 1. |
| 18 | 347 | *I appreciate that diagnoses within a set period were extended across time and visits (all encounter diagnoses on the day of diagnosis by any provider and diagnoses by same provider across next 21 days being examined for possible antibiotic appropriate diagnoses). In contrast, it appears that categorization of visit type (in-person vs non-in-person) was more rigid. For example, if a pharmacy called to clarify a prescription on the day of or day after an in-person visit prompting a telephone encounter with a new prescription, was this classified as a telephone encounter or associated with the preceding in-person encounter? Were these counted as two separate antibiotic prescriptions in two different settings, or only ultimately counted as a telephone encounter (with the prior in-person prescription perhaps cancelled in the EHR?)? Sensitivity analysis around these assumptions seem important to understand out-of-visit prescribing.*  In the situation that Reviewer 1 describes, we would have counted the patient as having two antibiotic prescriptions. This is because we did not count cancelled prescriptions as prescriptions. While considering the patient to have two prescriptions would result in double counting, any resulting bias is likely minimal, as only 2% of antibiotic prescriptions in our analysis were associated with another antibiotic prescription for the same patient within 1-3 days. We would not want to exclude this 2% from the analysis, as it is completely possible that the subsequent prescription within 1-3 days represents a legitimately separate prescription (e.g., owing to intolerance of the first prescription).  To clarify the manuscript, we added the following sentences: “Fifth, our approach assumed that antibiotic prescriptions on different days resulted from a separate episode of care, but this may not always be the case. For example, if a follow-up telephone visit by a patient or pharmacist to clarify an antibiotic written at an in-person visit resulted in a new antibiotic prescription, this would have been counted as two separate prescriptions. However, any resulting bias is likely minimal, as only 2% of antibiotic prescriptions had another antibiotic prescription in the subsequent 1 to 3 days.” |
| 6  16 | 120  297 | *It seems important to re-emphasize that during the study period “at least one diagnosis was required for an in-person encounter, but not for a telephone or patient portal encounter.” Thus I would hesitate to interpret the 42% not-in-person visits without a diagnosis as inappropriate prescribing, but rather inappropriate documentation enabled by different requirements within the EHR for different visit types, leaving the question of appropriateness wide open for these visits, and necessitating further examination in a EHR where/when diagnoses are required.*  We agree. To strengthen Reviewer 1’s point, we have made a few changes. First, COVID-19 billing rule changes did not require a telephone or patient portal encounter diagnosis, so we have deleted that phrase.  Second, to re-emphasize Reviewer 1’s point, we have added the sentence “The absence of a diagnosis may say more about inappropriate documentation than inappropriate antibiotic prescribing.” |
| 5 | 111 | *Given the inclusion of chronic antibiotic use as a category of interest, unclear why antibiotics for urinary tract infection prophylaxis were specifically excluded.*  We appreciate Reviewer 1 pointing out our lack of clarity. Functionally, we only excluded methenamine, an antibiotic essentially used solely for urinary tract infection prophylaxis and suppression. To clarify, we have replaced the entire phrase “…antibiotics most often used for urinary tract infection…” with “methenamine.” |
| 18 | 343 | *Would emphasize in methods and discussion/limitation that the specific antibiotic could be inappropriate for the specific diagnosis and yet still classified as “appropriate” in this schema.*  We appreciate Reviewer 1 picking up on this nuance and have added the following to the “leniency” statement in our Limitations paragraph: “…did not require the antibiotic class to be appropriate for a given condition….” |
| 13 | 231 | *Are dental prescriptions included in these data? Given the focus on global ambulatory prescribing, would be worth noting whether this setting is included or not.*  Thank you for asking for clarification about dental prescribing. Dentists are not included in our data. We added the following to a footnote in Table 1: “Northwestern Medicine does not provide dental care.” |

**Reviewer 2 Comments**	NB: Page and Line Numbers are from revised, tracked changes revision

| **Page** | **Line** | **Comment** |
| --- | --- | --- |
| General Comment | | *This is a very interesting topic, although clearly the world has changed since the study's end. Overall, I had only one major issue. The introduction is well reasoned.*  We appreciate the positive feedback and thoughts from Reviewer 2. |
| 5 | 111 | *I would clarify you didn't look at antibiotics only prescribed for prophylaxis such as methenamine; nitrofurantoin and bactrim may be much more commonly used for UTI prophy. Also, dapsone for example?*  Similar to Reviewer 1, we appreciate Reviewer 2 pointing our lack of clarity. Please see our response to Reviewer 1 in which we replaced the phrase in question with the single drug that was excluded, methenamine. |
| 6 | 120 | *I would assume that even post-COVID, patient portal encounters and many non-visit phone encounters do not require a billing code*  Reviewer 2 is correct. Because of study stopped before the COVID pandemic, this point is out of scope for the present analysis. As noted above, to clarify this point, we removed the phrase “prior to COVID-19 billing rule changes” from the passage in question. |
| 9 | 203 | *The methods are well-described. However, was there a reason specifically for not doing testing for statistical significance? I understand and appreciate the clinical significance. It just makes it quite difficult to say, "more likely to be..."*  With antibiotics being the unit of analysis, the large sample size (n = 714,057) would make clinically insignificant differences statistically significant. Rough calculations show that with alpha of 0.05 with our sample size, even near 50% prevalence (where power is lowest), we have 99% power to detect a 0.5% difference between groups. Although culturally accepted, the traditional statistical cut-off of p < 0.05 is simply a choice. We made an analogous choice that absolute differences of 5% are clinically significant and thus say something is a notable difference, or one association is “more likely” than another.  To clarify the manuscript, we have modified the beginning of the sentence in question to read “Because the large sample size makes clinically insignificant differences statistically significant…” |
| 18 | 339 | *The rate of patients receiving antibiotics for chronic conditions seems extremely high. Almost as high, in fact, as the total proportion of antibiotics prescribed outside of clinic visits. Although I'm not sure of research supporting this, it is certainly higher than in many large health systems. It is also odd that the most common chronic diagnoses include chronic sinusitis, COPD, and chronic rhinitis (and not, for example, trimethoprim-sulfamethoxazole for PJP prophylaxis). Chronic sinusitis and chronic rhinitis do not seem like conditions necessitating chronic antibiotics. COPD also seems like a very small minority of patients would require chronic antibiotics, as general use of chronic antibiotics goes against international guidelines in this condition. Should this just be "sometimes" rather than "chronic?" As these categories are mutually exclusive, the chronic condition results are somewhat misleading. The fact that not many of these antibiotics are prescribed over refills also makes me suspect that these antibiotics are not really being prescribed for chronic infections.*  *I would expand on some of these limitations in the discussion as well.*  We agree with Reviewer 2 we have been extremely lenient towards designating an antibiotic prescription as chronic. To clarify Reviewer 2’s points in the manuscript, we modified the sentence in question to be: “Fourth, in determining appropriateness, we erred on the side of leniency. For example, we considered all antibiotic prescribing for chronic conditions to be acceptable (e.g., emphysema, chronic sinusitis, and chronic rhinitis) even though the prescribing may not have been clinically appropriate.” We also added, “Thus, our study’s estimate of inappropriate prescribing is undoubtedly a lower bound.” |
