# Supplementary material for: Prevalence and appropriateness of in-person versus not-in-person ambulatory antibiotic prescribing in an integrated academic health system: A cohort study
Source: PLoS One. 2023 Jul 27;18(7):e0289303. doi: 10.1371/journal.pone.0289303 (PMC10374053; doi:10.1371/journal.pone.0289303)
Supplement: Attachment — Submitted filename: PONE-D-23-01039R2, Response to Reviewers.docx [file pone.0289303.s004.docx]

**Journal Requirements**	NB: Page and Line Numbers are from revised, tracked changes revision

| **Page** | **Line** | **Comment** |
| --- | --- | --- |
| General Comment | | *Please review your reference list to ensure that it is complete and correct. If you have cited papers that have been retracted, please include the rationale for doing so in the manuscript text, or remove these references and replace them with relevant current references. Any changes to the reference list should be mentioned in the rebuttal letter that accompanies your revised manuscript. If you need to cite a retracted article, indicate the article’s retracted status in the References list and also include a citation and full reference for the retraction notice.*  We have reviewed the reference list for completeness and correctness. None of the cited references have been retracted. |

**Reviewer 2 Comments**	NB: Page and Line Numbers are from revised, tracked changes revision

| **Page** | **Line** | **Comment** |
| --- | --- | --- |
|  |  | *Thank you for addressing these comments. The article is now clearer. However, I have the most minor of revisions to request. On reviewing again, I am realizing that the distinction between adult vs pediatric patients (and internal medicine vs family medicine vs pediatrics) is unclear. Were pediatric patients included as well as adults?*  Thank you for pointing out our lack of clarity. We included patients regardless of age and we included all specialties. The primary care specialties (including family medicine, internal medicine, and pediatrics) were grouped together.  To clarify this in the manuscript, we modified the sentence “We included antibiotics prescribed **by clinicians of all specialties** to patients at an ambulatory clinic, regardless of **patient age**; whether the patient lived at home, at a care facility, or elsewhere**; or any other patient factor**. |
